# Adult-Onset Vitelliform Macular Dystrophy caused by BEST1 p.Ile38Ser Mutation is a Mild Form of Best Vitelliform Macular Dystrophy

**DOI:** 10.1038/s41598-017-09629-9

**Published:** 2017-08-22

**Authors:** Ikhyun Jun, Joon Suk Lee, Ji Hwan Lee, Christopher Seungkyu Lee, Seung-il Choi, Heon Yung Gee, Min Goo Lee, Eung Kweon Kim

**Affiliations:** 1 The Institute of Vision Research, Department of Ophthalmology, Yonsei University College of Medicine, Seoul, Korea; 2 Corneal Dystrophy Research Institute, Yonsei University College of Medicine, Seoul, Korea; 3 Department of Pharmacology and Brain Korea 21 PLUS Project for Medical Sciences, Yonsei University College of Medicine, Seoul, Korea; 4 Severance Biomedical Science Institute, Yonsei University College of Medicine, Seoul, Korea

## Abstract

Adult-onset vitelliform macular dystrophy (AVMD) is a common and benign macular degeneration which can be caused by *BEST1* mutation. Here, we investigated the clinical characteristics associated with a newly identified BEST1 mutation, p.Ile38Ser and confirmed the associated physiological functional defects. The 51-year-old patient presented bilateral small subretinal yellow deposits. Consistent with AVMD, the corresponding lesions showed hyperautofluorescence, late staining in fluorescein angiography, and subretinal hyper-reflective materials in spectral-domain optical coherence tomography. Genetic analysis demonstrated that the patient presented with a heterozygous p.Ile38Ser BEST1 mutation. Surface biotinylation and patch clamp experiments were performed in transfected HEK293T cells. Although, the identified BEST1 mutant maintains normal membrane expression, p.Ile38Ser mutant showed significantly smaller currents than wild type (WT). However, it showed larger currents than other BEST1 mutants, p.Trp93Cys, causing autosomal dominant best vitelliform macular dystrophy (BVMD), and p.Ala195Val, causing autosomal recessive bestrophinopathy (ARB). The cells expressing both WT and each BEST1 mutant showed that the functional defect of p.Ile38ser was milder than that of p.Trp93Cys, whereas combination of p.Ala195Val with WT showed good current. We identified and described the phenotype and *in vitro* functions of a novel *BEST1* mutation causing AVMD. AVMD induced by p.Ile38Ser BEST1 mutation is a mild form of BVMD.

## Introduction

Bestrophin-1 (*BEST1*, also known as *VMD2*) was first identified in Best vitelliform macular dystrophy (BVMD, Best disease), a disease associated with *BEST1* mutations^1–3^. BEST1 is predominantly expressed in the basolateral membrane of the retinal pigment epithelium (RPE)^4^. X-ray structure of chicken BEST1, sharing 74% of sequence homology with human BEST1, indicated that BEST1 forms a homo-pentamer and functions as a Ca^2+^-activated chloride (Cl^−^) channel (CaCC)^5^. Moreover, BEST1 appears to function as a regulator of intracellular calcium signaling^6^. More than 200 mutations of this gene have been reported to generate various ocular diseases, which are collectively referred to as bestrophinopathy. Bestrophinopathy includes at least five clinically distinct categories, BVMD, adult-onset vitelliform macular dystrophy (AVMD), autosomal recessive bestrophinopathy (ARB), autosomal dominant vitreoretinochoroidopathy (ADVIRC), and retinitis pigmentosa (RP)^2, 3^.

AVMD is one of the most common forms of macular degeneration^7^. The age of AVMD onset is highly variable, but patients have a tendency to remain asymptomatic until the fifth decade^3, 7^. The clinical characteristics of AVMD are relatively benign, including a small subretinal vitelliform macular lesion, a slower progression of disease, and a slight deterioration in electrooculography (EOG)^3, 7^. In some cases, AVMD is associated with autosomal dominant inheritance^8, 9^, with mutations in *PRPH2* ^10^, *BEST1* ^11^, *IMPG1* ^12^, or *IMPG2* ^13^. Fluctuating expression and decreased penetrance could partly explain the scarcity of a clear inheritance pattern.

Allikmets and colleagues published the first report describing the BEST1 mutation, p.Ala146Lys, in a patient with AVMD in 1999^14^. The authors screened for *BEST1* mutation in patients with age-related macular degeneration (AMD) and other maculopathies and concluded that *BEST1* mutations could cause various disease phenotypes. Subsequently, four additional *BEST1* mutations, including p.Thr6Pro, p.Arg47His, p.Ala243Val, and p.Asp312Asn, were identified in patients with AVMD^11^. Since then, no further mutations in *BEST1* have been identified by genetic screening of patients with AVMD^15–19^, although late onset best macular dystrophy with *BEST1* mutations has been investigated^20^.

Currently, because of similar clinical characteristics, AVMD induced by *BEST1* mutations is considered a late-onset and mild form of BVMD^2, 3, 7^, although there is no case report or no combined experimental evidence. No study has investigated the direct association between *BEST1* mutation and AVMD pathophysiology. In this study, we present an AVMD case presenting with a novel *BEST1* mutation, p.Ile38Ser, and propose that *BEST1* mutation is the causative factor of AVMD based on molecular and electrophysiological *in vitro* experiments.

## Results

### Clinical findings

**Figure 1:**
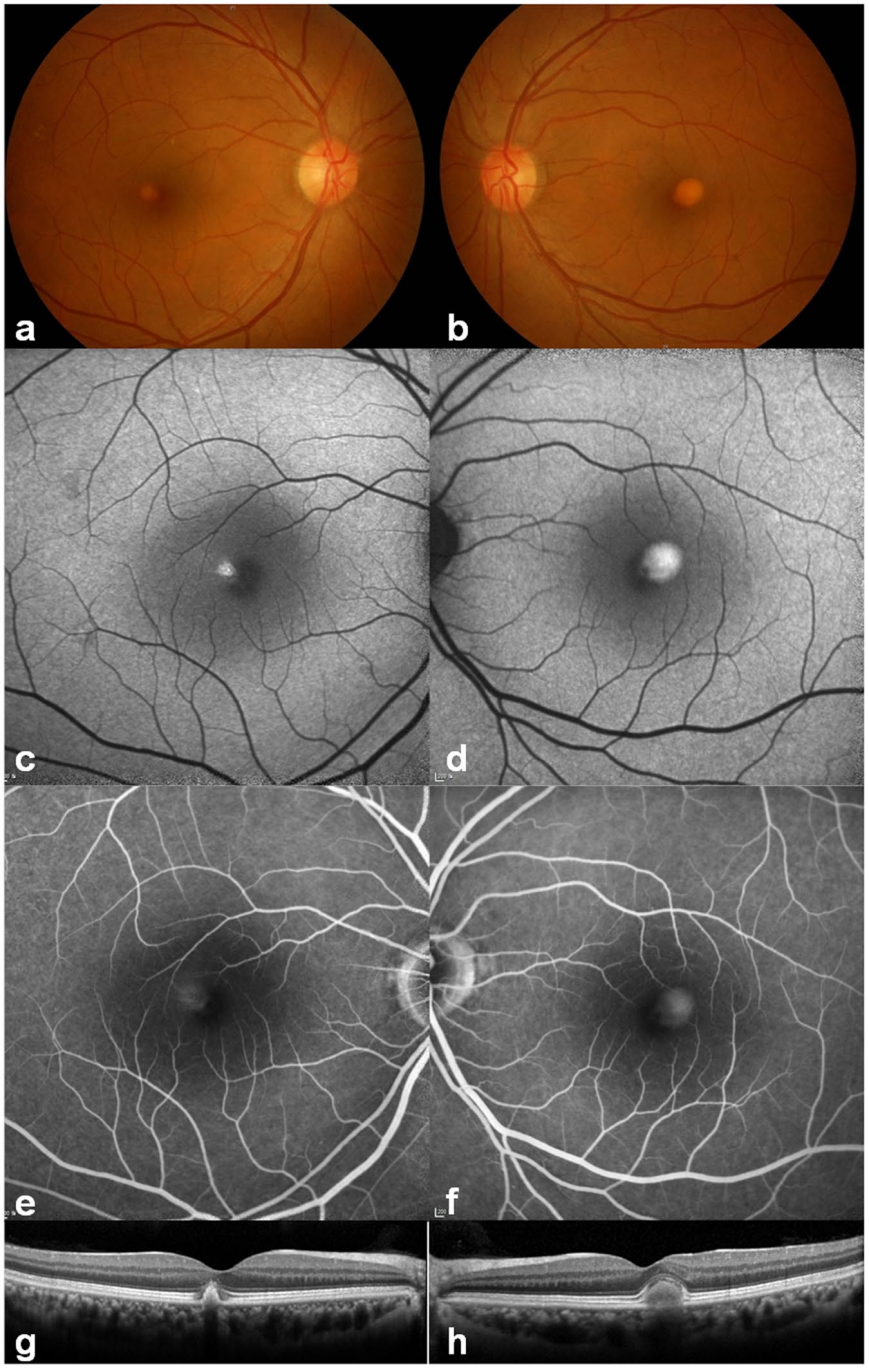
Clinical features of the patient. **(a**,**b)** Color fundus photographs showing bilateral, round subretinal yellowish deposits (vitelliform) in the posterior pole. **(c**,**d)** Blue light autofluorescence imaging showed intense hyper-autofluorescence at the corresponding yellowish deposit lesions. **(e**,**f)** Corresponding lesions showed diffuse hyperfluorescence in the late-phase images of fluorescein angiography. **(g**,**h)** Horizontal spectral-domain optical coherence tomography images through fovea showed well demarcated subretinal hyper-reflective material in the affected lesion.

The demographic and clinical details of the patient with AVMD are summarized in Table 1. A 51-year-old man was referred to our hospital because of retinal lesions in both eyes detected during health checkup. His best-corrected visual acuity was 20/20 in the right eye and 20/22 in the left eye. The patient was slightly myopic and his intraocular pressure was within the normal range. No specific sign was detected in his anterior segment of the eyes. The fundus showed small subfoveal yellowish deposits and some drusens on the right eye, and one-fourth of the disc area sized subfoveal yellowish deposits on the left eye (Fig. 1a and b). Increased autofluorescence at the yellowish subfoveal deposits was detected by blue-light autofluorescence imaging (Fig. 1c and d). The fluorescein angiography showed staining of the vitelliform material corresponding to the area of retinal lesion (Fig. 1e and f). SD-OCT showed well demarcated and dome-shaped subretinal hyper-reflective material in the affected lesion (Fig. 1g and h). Full-field ERG showed the normal range of scotopic and photopic responses. The EOG showed a decreased light rise (Arden ratio, 1.6 (OD), 1.4 (OS)). His clinical manifestations did not change during the 2-year follow-up.

**Table 1:** Demography, Clinical Findings, and In Silico Analysis of the BEST1 mutation.

Sex	Age (yr)	Decimal Visual Acuity (Spherical Equivalent, D)		ERG		EOG, Arden Ratio		Nucleotide change^a^	Amino acid change	Amino acid sequence conservation^b^	Frequencies in the dbSNP database^c^	Frequencies in the ExAC database^d^	Frequencies in the NBK database^e^	PP2^f^	MT^g^	PRO-VEAN^h^	SIFT^i^
		OD	OS	OD	OS	OD	OS										
M	51	1.0 (−0.375)	0.9 (−0.25)	WNL	WNL	1.6	1.4	c.113T > G	p.Ile38Ser	*D. rerio*	ND	ND	ND	PD (0.999)	DC (1.000)	Del (−3.27)	Dam (0.033)

Abbreviations are as follows: D, diopter; Dam, damaging; DC, disease causing; Del, deleterious; EOG, electro-oculogram; ERG, electroretinogram; M, male; ND, no data or DNA available; OD, right eye; OS, left eye; PD, probably damaging; PP2, PolyPhen-2 prediction score Humvar; PROVEAN, Protein Variation Effect Analyzer; SIFT, Sorting Intolerant from Tolerant; SNP, single nucleotide polymorphism; WNL, within normal limits; yr, years. ^**a**^cDNA mutations are numbered according to human cDNA reference sequence NM_004183; **+**1 corresponds to the A of ATG translation initiation codon. ^b^Amino acid residue is continually conserved throughout evolution including the species as indicated. ^c^dbSNP database (http://www.ncbi.nlm.nih.gov/SNP). ^d^ExAC browser (http://exac.broadinstitute.org/). ^e^National Biobank of Korea, the Centers for Disease Control and Prevention. ^f^PolyPhen-2 prediction score HumVar ranges from 0 to 1.0; 0 = benign, 1.0 = probably damaging (http://genetics.bwh.harvard.edu/pph2/). ^g^Mutation taster (http://www.mutationtaster.org/). ^h^PROVEAN (http://provean.jcvi.org/index.php). ^i^SIFT (http://sift.jcvi.org/).

### Genetic findings

**Figure 2:**
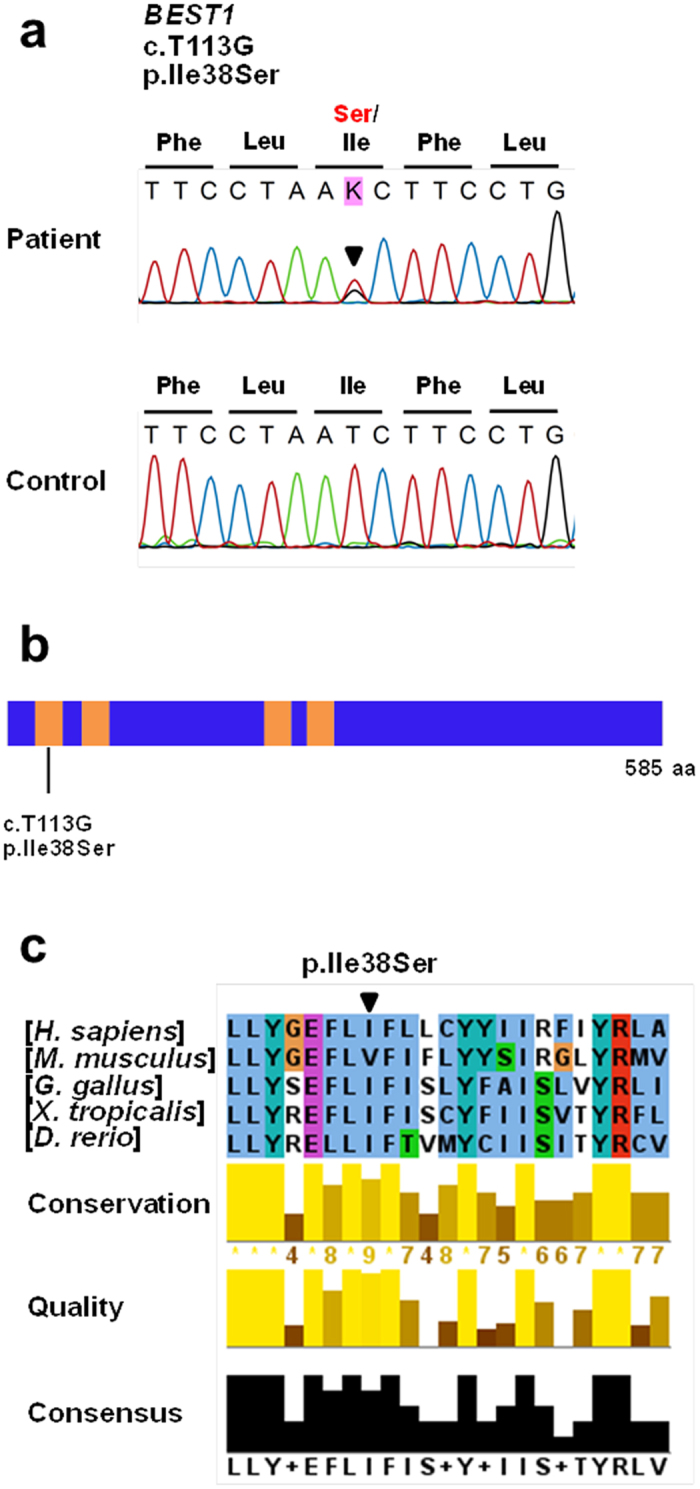
Genetic analysis of the patient identified a novel BEST1 mutation, p.Ile38Ser. **(a)** Sequencing traces of the mutation and wild type control. Altered nucleotide and amino-acid changes are indicated above the sequence traces. **(b)** Domain structure of BEST1. The transmembrane (TM) domains are depicted by orange colored bars. The BEST1 mutation is located in the TM1 domain. **(c)** Partial protein alignment of BEST1 TM1 domain showed evolutionary conservation of the identified missense changes.

A heterozygous c.T113G missense mutation (p.Ile38Ser) was detected in the *BEST1* gene of the patient, suggesting the autosomal dominant inheritance of this mutation (Fig. 2a). We also examined *PRPH2*, *IMPG1*, and *IMPG2*, which are other known genes for AVMD, by Sanger sequencing. Several nonsynonymous variants were detected (Supplementary Table S1), but all of them are reported as single nucleotide polymorphism (SNP), of which minor allele frequencies are greater than 1%; therefore, they are not likely to be disease-causing. The BEST1 p.Ile38Ser mutation has not been previously reported in patients with *BEST1*-associated disease, or in SNP databases such as dbSNP or Exome Aggregation Consortium (ExAC) (Table 1). This mutation is not found in the National Biobank of Korea (NBK) data of whole genome sequencing of 397 healthy individuals or in our in-house whole exome sequencing data (59 subjects) described in our previous study^21^. The amino acid residue affected by this missense mutation is located in the transmembrane (TM) domain 1, which is well conserved throughout evolution down to *Danio rerio* (Table 1 and Fig. 2b and c). Moreover, several prediction algorithms suggested that p.Ile38Ser mutation has high disease-causing probability (Table 1).

### *In vitro* study

**Figure 3:**
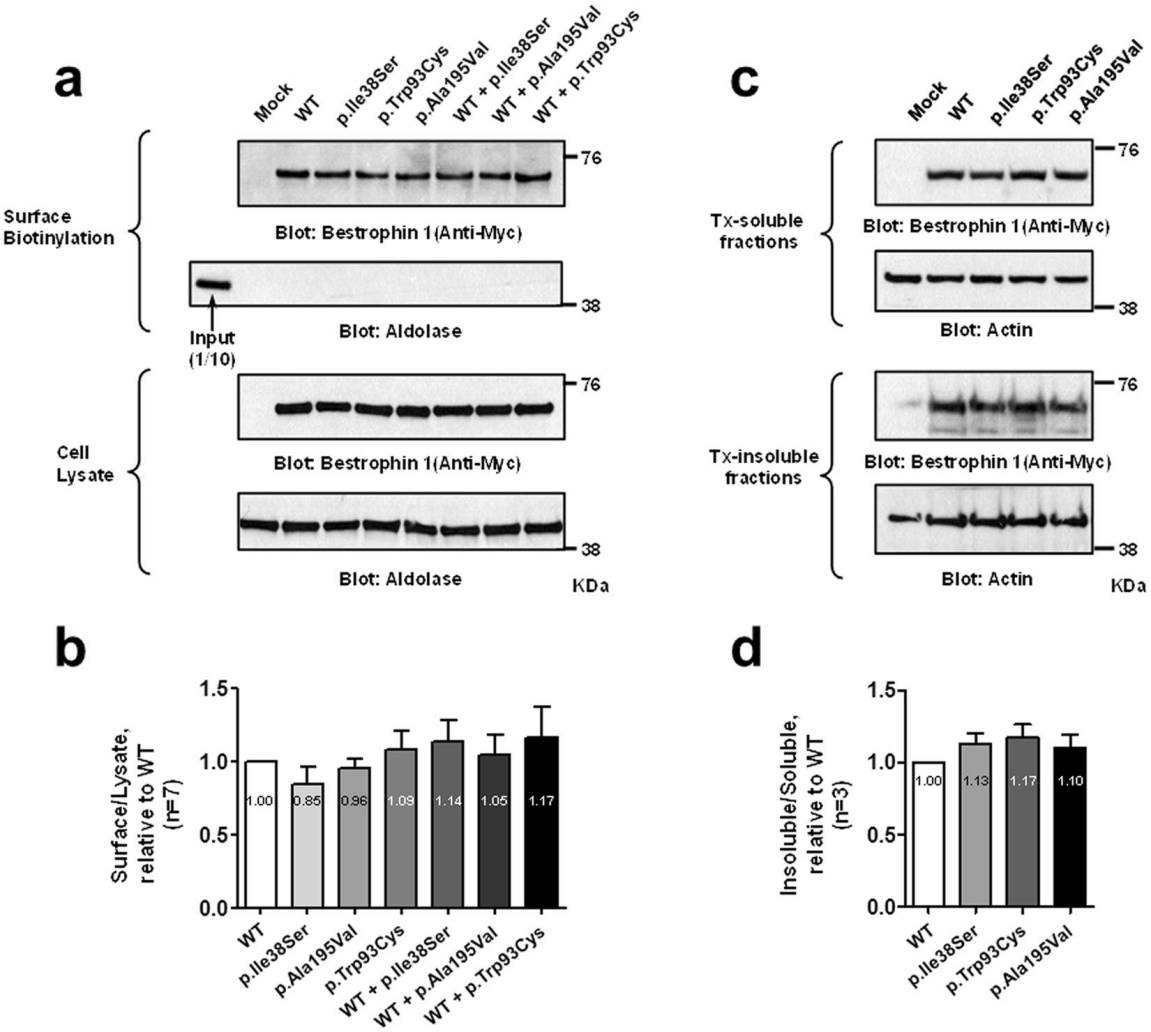
BEST1 p.Ile38Ser mutation does not interfere with membrane expression. HEK293T cells were transfected with plasmids expressing wild type and mutant hBEST1. **(a)** Surface biotinylation assays of hBEST1 were performed. **(b**) Quantitation of multiple experiments shows that the membrane expression was not different among groups by ANOVA (P = 0.589). Fold changes relative to WT are provided. **(c)** Detergent solubility assay of wild type and mutant hBEST1. **(d)** Quantification of multiple experiments shows that the detergent solubility was not different among the groups by ANOVA (P = 0.300). Fold changes relative to WT are provided. Western blots were cropped to show specific bands only. For uncropped blots see Supplementary Fig. S2.

We conducted *in vitro* experiments to examine functional defects caused by this mutation. We evaluated the membrane expression of BEST1 p.Ile38Ser mutant. We performed surface biotinylation assays, which can detect proteins only on the plasma membrane, after transfection of wild type (WT) and mutant h*BEST1* clones into HEK293T cells (Figs. 3a and b). BEST1 p.Ile38Ser mutant is expressed in the membrane as WT and other mutants, p.Trp93Cys and p.Ala195Val, which were previously shown to be expressed in the membrane (Fold change; WT = 1; p.Ile38Ser = 0.85 ± 0.12; p.Ala195Val = 0.96 ± 0.07; p.Trp93Cys = 1.09 ± 0.13; WT + p.Ile38Ser = 1.14 ± 0.15; WT + p.Ala195Val = 1.05 ± 0.14; WT + p.Trp93Cys = 1.17 ± 0.21; P = 0.589)^22^. Membrane expression of WT and p.Ile38Ser was also confirmed by immunocytochemistry (Supplementary Fig. S1). WT and mutant BEST1 exhibited no difference in the detergent solubility assay, which evaluates intracellular aggregation (Figs. 3c and d, Fold change; WT = 1, p.Ile38Ser = 1.13 ± 0.08; p.Trp93Cys = 1.17 ± 0.09; p.Ala195Val = 1.10 ± 0.09; P = 0.300).

**Figure 4:**
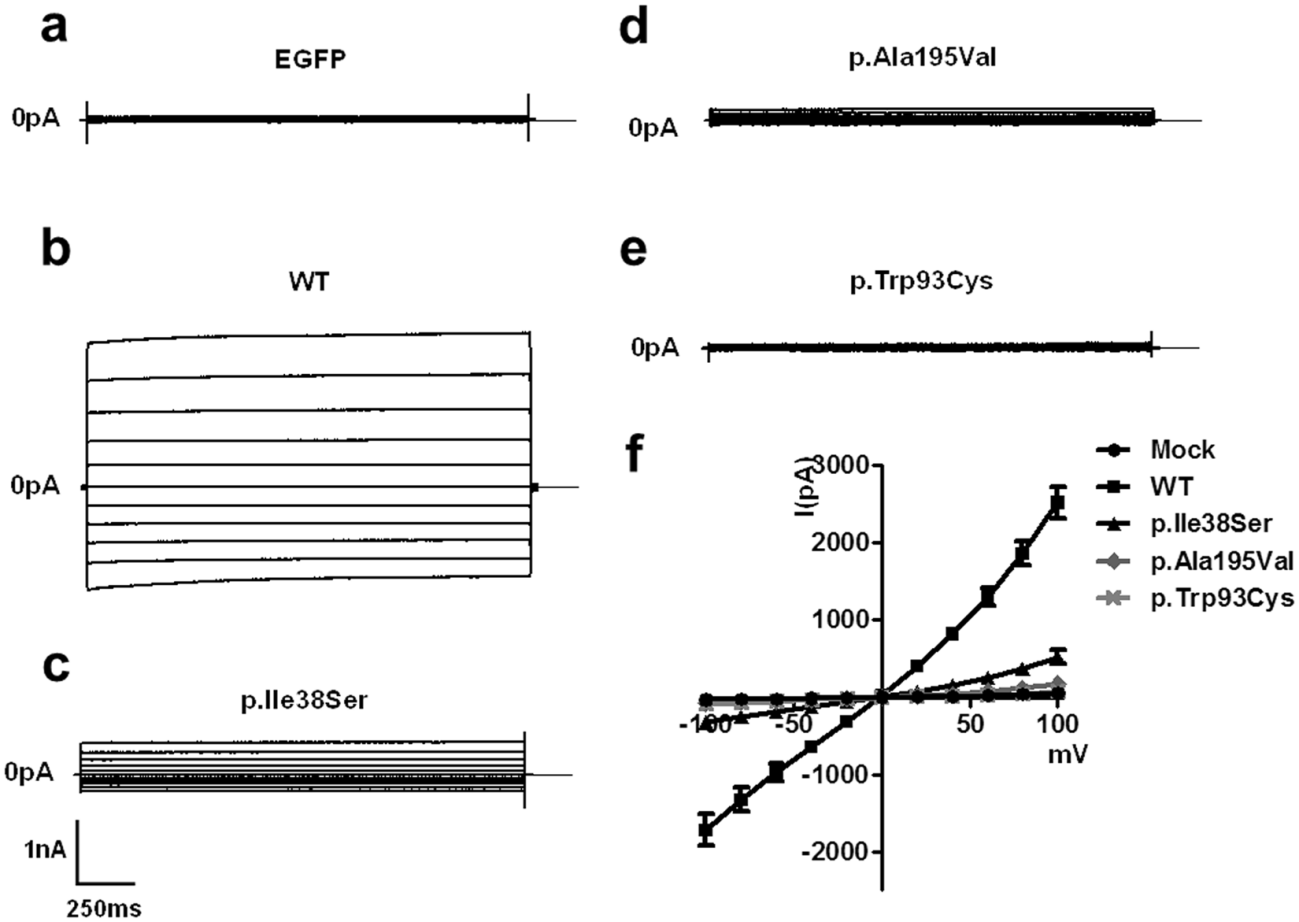
p.Ile38Ser BEST1 mutant shows smaller currents compared to wild type (WT) BEST1 and bigger currents compared to p.Ala195Val or p.Trp93Cys mutants. **(a–e)** Representative traces of HEK293T cells transfected with EGFP alone **(a)**, WT **(b)**, p.Ile38Ser **(c)**, p.Ala195Val **(d)**, or p.Trp93Cys **(e)** BEST1. Voltage was stepped from a holding potential of 0 mV to between −100 and +100 mV in 20 mV steps. Step duration was 2000 ms. **(f)** Current-voltage relationship of mock, WT, and mutant BEST1. Data are presented as mean ± SEM.

Subsequently, we measured whole-cell currents of the WT BEST1 and mutants after activation by calcium in transfected HEK293T cells (Fig. 4, Supplementary Table S2). WT BEST1 generated significantly larger currents than those observed in the mock-transfected HEK293T cells as reported in previous studies (P < 0.001)^22, 23^. In addition, the currents in the cells expressing p.Ala195Val and p.Trp93Cys were significantly smaller than those in cells expressing WT, consistent with previous studies (Supplementary Table S2)^22^. p.Ile38Ser mutant showed a smaller current, about 20% of WT BEST1, which was statistically significant (P < 0.001). However, the current of p.Ile38Ser was approximately 7 times larger than that of the p.Trp93Cys mutant, although it did not reach statistical significance (Supplementary Table S2). These data suggest that the p.Ile38Ser mutation may result in a mild form of bestrophinopathy.

**Figure 5:**
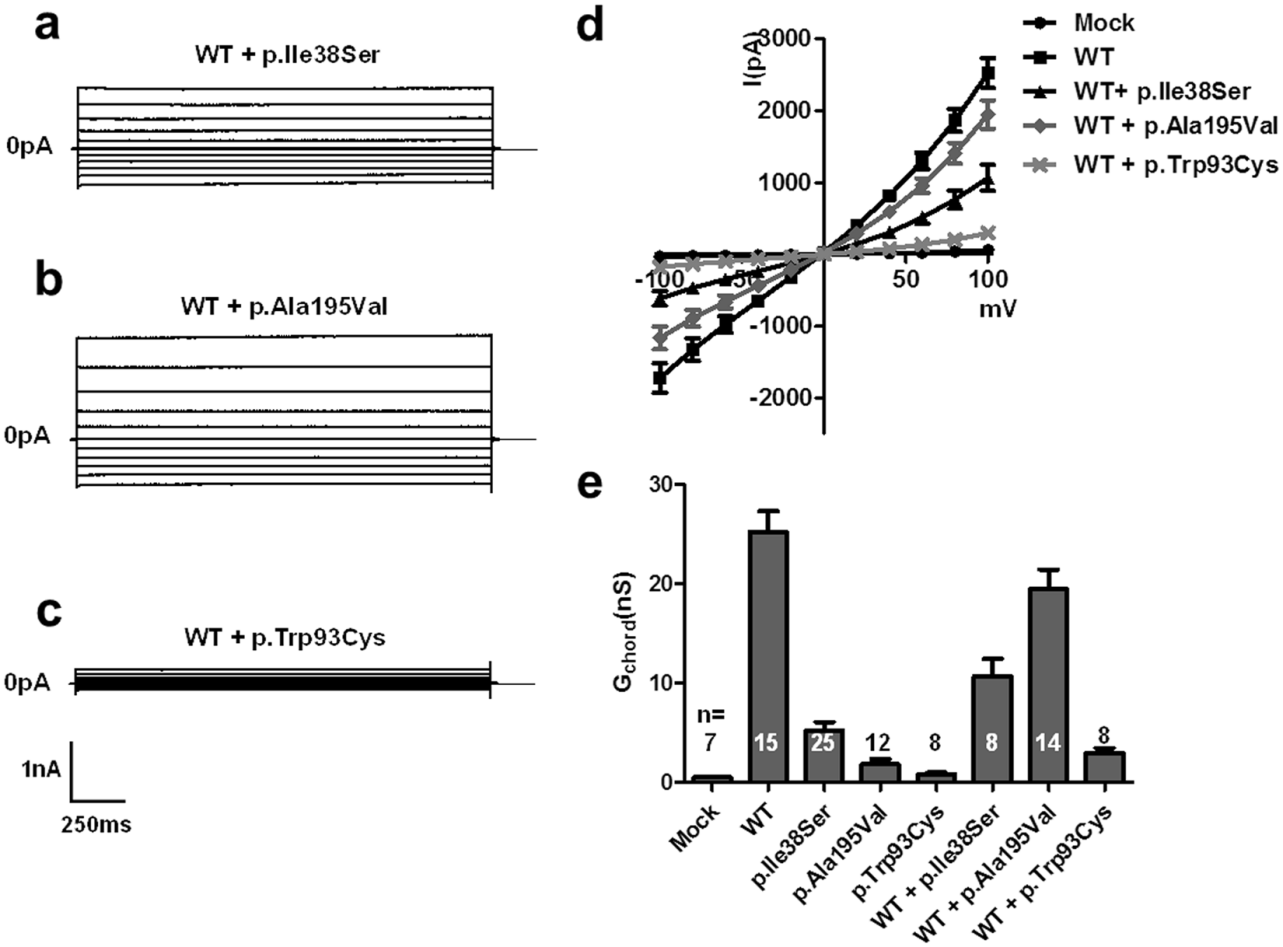
p.Ile38Ser mutation induces mild deterioration of protein function. **(a–c)** Representative traces of HEK293T cells transiently transfected with wild type (WT) BEST1 plus either p.Ile38Ser **(a)**, p.Ala195Val **(b)**, or p.Trp93Cys **(c)**. **(d)** Current-voltage relationship of mock, WT, and co-transfection of WT and mutant BEST1. **(e)** The mean outward chord conductance (G_chord_) in transfected HEK293T cells, calculated over 0 mV to +100 mV. Co-expression of WT and p.Ile38Ser BEST1 results into approximately one-third of the currents of WT BEST1, while p.Trp93Cys generated very small currents. The p.Ala195Val mutant, an autosomal recessive type, elicited large currents when co-expressed with WT BEST1. Data are presented as mean ± SEM. (Conductance values are provided in Supplementary Table S2. WT *vs*. WT + p.Ile38Ser, p < 0.01; WT + p.Ile38Ser *vs*. WT + p.Ala195Val, p < 0.001; WT + p.Ile38Ser *vs*. WT + p.Trp93Cys, p < 0.01 by ANOVA followed by Newman-Keuls multiple comparison test, details are provided in Supplementary Table S2).

Given that the patient was heterozygous for this mutation, we evaluated BEST1 function in HEK293T cells after co-expression of WT and p.Ile38Ser BEST1 (Fig. 5, Supplementary Table S2). Cells co-expressing WT and p.Ile38Ser BEST1 showed approximately one-third of the currents observed in cells expressing WT BEST1 (P < 0.001). HEK293T cells co-transfected with plasmids expressing WT BEST1 and p.Ala195Val, known as an autosomal recessive mutant^22–24^, generated 80% of the current observed in cells expressing WT BEST1 (Fig. 5, Supplementary Table S2). Meanwhile, co-expression of an autosomal dominant-type mutant, p.Trp93Cys, and WT BEST1 resulted in the activation of diminutive currents as previously reported (Fig. 5, Supplementary Table S2)^22^. These findings suggest that p.Ile38Ser mutation is probably associated with autosomal dominant bestrophinopathy, presenting a milder phenotype than the classical BVMD mutant, p.Trp93Cys. Additionally, a threshold current of disease occurrence associated with *BEST1* mutation might be located between the values of p.Ala195Val and p.Ile38Ser.

#### Structure analysis

**Figure 6:**
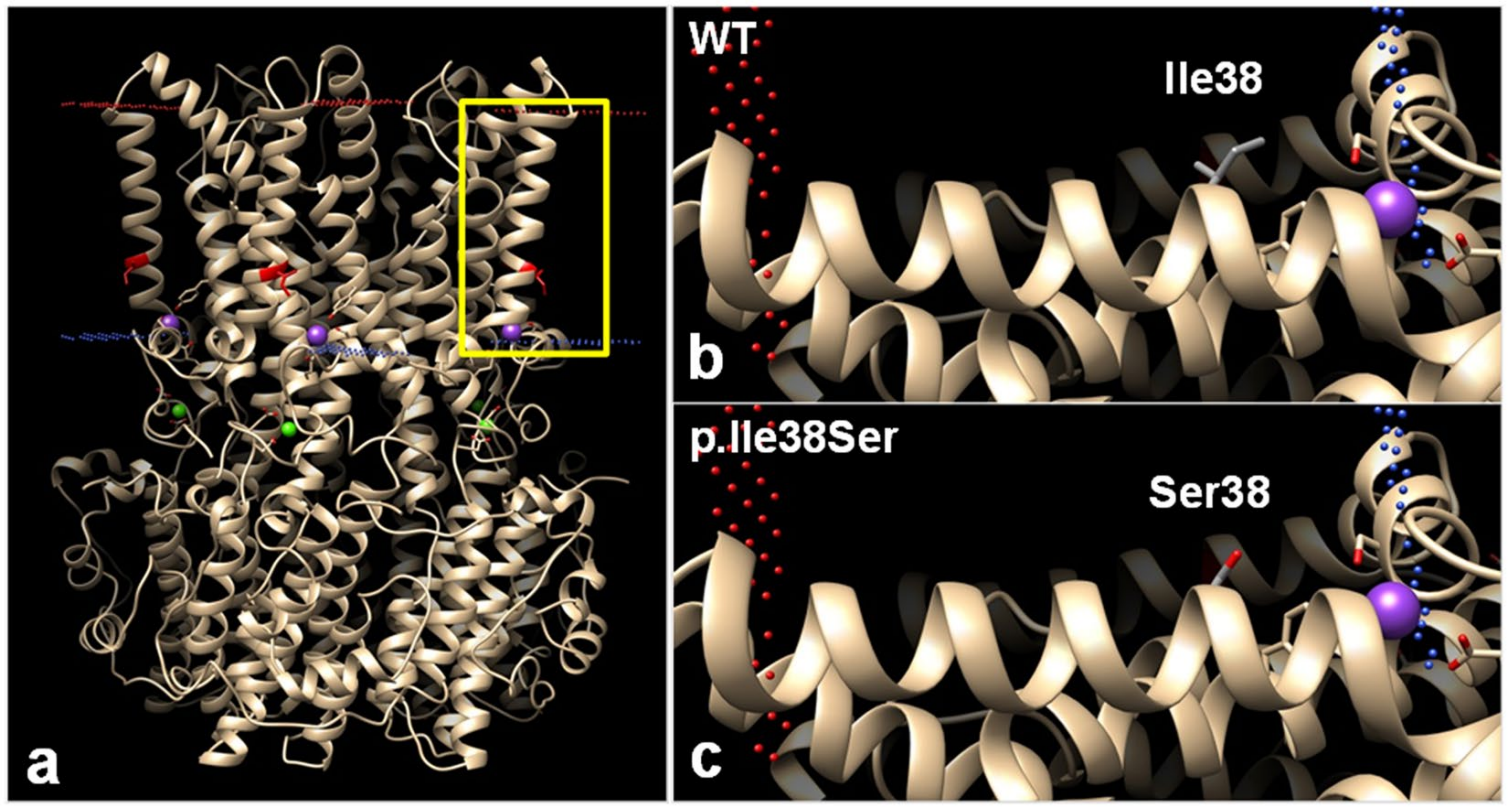
Predicted computational tertiary structure of wild-type (WT) and p.Ile38Ser hBEST1 proteins. 4RDQ crystal structure was used as template. **(a)** Homopentameric structure of WT BEST1 full structure. Red dots represent outer membrane border and blue dots represent intracellular membrane border. Isoleucine 38 position, which is located in the transmembrane (TM) domain 1, is highlighted in red. The yellow box region was magnified in **(b)** and **(c)**. **(b)** The side chain of Ile38 in the TM1 domain was described, which consisted of a hydrophobic carbon chain (gray). The side chain protrudes toward the lipid membrane. **(c)** The side chain of Ser38 presents hydrophilic oxygen residue (red), which may generate a repulsive force with the hydrophobic lipid membrane.

A 3-dimensional protein modeling was performed to examine structural defects caused by the p.Ile38Ser mutation (Fig. 6). The side chain of Ile38 in TM1 domain protrudes toward the lipid membrane (Fig. 6b) and is composed of hydrophobic carbon, whereas the side chain of p.Ile38Ser mutant has oxygen residues, which may generate a repulsive force with the lipid membrane (Fig. 6c). Therefore, the mutation can affect the protein structure and function.

## Discussion

AVMD is typically known to present with central round yellow deposit (<1 disc diameter), hyper-autofluorescent lesion, late staining in fluorescein angiography, and subnormal to normal EOG^7^. Our patient also presented a bilateral small hyper-autofluorescent, vitelliform macular lesion, and subnormal EOG (Arden ratio 1.6 for right eye, and 1.4 for left eye), consistent with AVMD. Despite these fundoscopic pathologies, he presented no symptom, and his visual acuity was good in both eyes. The patient was first examined at age 51, and the clinical phenotypes did not change during the 2-year follow-up. Interestingly, the left eye showed a larger lesion than the right eye, consistent with the lower visual acuity and Arden ratio in EOG of the left eye.

The BEST1 p.Ile38Ser mutation identified in this study is located in the TM1 domain, which is well conserved in all vertebrates (Table 1 and Fig. 2). The well conserved regions of a gene imply their involvement in physiological functions and mutations can render the gene defective. Prediction of pathogenicity for this mutation also demonstrated a high likelihood of deterioration (Table 1). The side-chain of isoleucine is hydrophobic and protrudes into the lipid bilayer^5^, but the substituted serine contains a hydrophilic hydroxyl group in the side chain (Fig. 6). If the hydroxyl group protrudes, the repulsive force between the hydrophilic side chain and hydrophobic tails of phospholipids can be generated, causing structural disturbance.

Because the clinical features of AVMD are similar to those of early stage BVMD and the age of BVMD onset is highly variable, many groups have suggested that AVMD is a mild form of BVMD and presents the same spectrum^2, 3^. However, physiological and experimental evidence is insufficient and unsatisfactory. We conducted *in vitro* experiments to directly investigate the association between the novel variant of BEST1 (p.Ile38Ser) and AVMD. The membrane expression of the BEST1 p.Ile38Ser mutant was comparable to that of WT (Fig. 3) and similar to the expression of other autosomal dominant *BEST1* mutations^22, 25–27^. The defect associated with p.Ile38Ser mutation is not likely to be due to deterioration of membrane localization. In fact, the pathogenesis of autosomal dominant *BEST1* mutation mostly results from the assembly of a heteropentameric structure together with defective mutant and normal BEST1, which results in impairment of CaCC function^5, 28^. Therefore, intact membrane expression of BEST1 mutant can be associated with the development of the disease. The detergent solubility assay showed no difference between WT and p.Ile38Ser, indicating that the pathogenesis of p.Ile38ser is not related with protein insolubility.

Next, we evaluated the current activation of the BEST1 mutants by whole cell patch-clamp recording. The p.Ile38Ser mutant generated reduced calcium-activated currents, which verifies that this mutation is the causative factor of the disease (Fig. 4). The current generated by the p.Ile38Ser was bigger than those of p.Trp93Cy and p.Ala195Val, possibly explaining the mild clinical features of the patient with p.Ile38Ser mutation. Furthermore, co-expression of WT and p.Ile38Ser produced a small, but consistent, degree of currents, approximately 30% of that produced by WT BEST1, whereas p.Trp93Cys, a variant of BVMD, and WT elicited almost no current (Fig. 5). These data are consistent with subnormal to normal values of EOG in our patient and patients with AVMD and absent light rise in patients with BVMD, because EOG reflects a Cl^−^ conductance in the basolateral membrane of the RPE where BEST1 is localized^4, 29–31^. In addition, p.Ala195Val, an autosomal recessive variant, showed much bigger currents than p.Ile38Ser when co-transfected with WT BEST1 (Fig. 5). Thus, we speculate that p.Ile38Ser mutation presents autosomal dominant inheritance with dominant negative character.

Yu and colleagues investigated other BEST1 variants associated with AVMD, p.Ala146Lys, p.Ala243Val, and p.Asp312Asn^25, 26^. These variants are also expressed on the cell surface membrane. Cells transfected with these variants alone presented very small calcium-activated currents, and those of cells co-transfected with plasmids expressing WT BEST1 and each variant were moderately increased, similarly to the p.Ile38Ser mutant. Additionally, the EOG values of patients presenting these mutations were normal or slightly below baseline^11^. These previous data support the hypothesis that AVMD is a mild form of BVMD. Furthermore, some *BEST1* mutations causing BVMD are associated with variable clinical features regarding age at onset, lesion size, and EOG results^20^. Such variable phenotypes of bestrophinopathy result from variable expression patterns and reduced penetrance of BEST1. Therefore, BEST1 activity as a calcium-activated Cl^-^ channel, can determine the seriousness of the disease. We suggest that the degree of functional defect resulting from a certain *BEST1* mutation may dictate the phenotype as BVMD or AVMD.

This study has some limitations. We only identified one *BEST1* mutation causing AVMD. Moreover, the patient refused clinical evaluation and genetic analysis of his family members. Hence, we were unable to trace his pedigree. We only confirmed a modest functional defect of p.Ile38Ser mutant, although several BEST1 mutants causing AVMD are present. Nonetheless, this study is valuable, because it is the first study linking AVMD clinical features and molecular patho-mechanisms caused by a BEST1 mutation. Furthermore, our results suggest that increasing ion channel activity of BEST1 mutants could be a treatment option for bestrophinopathy, including BVMD. If we can improve the function of BEST1 mutant to one-third that of WT, BVMD, which causes visual loss, may be shifted to AVMD, which is benign. As for Ivacaftor, a CFTR potentiator, which has become a cornerstone drug for the treatment of cystic fibrosis^32, 33^, the development of a BEST1 potentiator can be a paradigm shift for the treatment of bestrophinopathy and even provide a complete cure. To examine more physiological pathomechanisms of BEST1 mutant, the experiments in RPE cells are needed in the future studies.

In this study, we conducted a comprehensive clinical, genetic, and molecular evaluation of AVMD associated with a heterozygous mutation in *BEST1*. *BEST1* mutation, detected in AVMD, showed intermediate deterioration of protein function. *In vitro* functional experiments showed that p.Ile38Ser is one of the *BEST1* autosomal dominant mutations associated with AVMD. This study establishes a connection between clinical phenotypes and AVMD pathogenesis at the molecular level. The hypothesis that AVMD is a mild form of BVMD and presents the same spectrum is verified by molecular and physiological experiments.

## Methods

### Clinical investigation

This study was approved by the Institutional Review Board of the Yonsei University College of Medicine (IRB No. 4-2017-0097) and followed the tenets of the Declaration of Helsinki. Written informed consent was obtained from the participant. A patient with AVMD was investigated in this study. The patient underwent a detailed ophthalmological examination, including best-corrected visual acuity, intraocular pressure measurement, slit-lamp examination, indirect ophthalmoscopy, fundus photography, fundus autofluorescence imaging, spectral-domain optical coherence tomography (SD-OCT), fluorescein angiography, and full-field electroretinography (ERG), and EOG. Full-field ERG and EOG were performed according to the guidelines of the International Society for Clinical Electrophysiology of Vision (www.iscev.org). The normal range of the Arden ratio of the EOG (ratio of the light peak to the dark trough) is more than 1.8 for our laboratory.

### Genetic analysis

Genetic analysis was performed as reported previously^22^. Genomic DNA was isolated from the subject’s blood using QIAamp RNA Blood Mini Kit (Cat. No. 51106, QIAGEN, Hilden, Germany). Each exon of the *BEST1* gene was amplified from genomic DNA by polymerase chain reaction (PCR) using the intronic oligonucleotide primers and PCR conditions described previously^34^. Each exon of the PRPH2, IMPG1, and IMPG2 genes was also amplified from genomic DNA by PCR using the intronic oligonucleotide primers (Supplementary Table S3). Each PCR was performed by using Maxime PCR PreMix (Cat. No. 25167, iNtRON Biotech., Seoul, Korea). PCR products were analyzed by direct sequencing using an Applied Biosystems (ABI) 3730 DNA sequencer (ABI, Foster City, CA, USA).

We examined the most recent versions of dbSNP (http://www.ncbi.nlm.nih.gov/SNP/), exome variant server (EVS; http://evs.gs.washington.edu/EVS/), and exome aggregation consortium (ExAC; http://exac.broadinstitute.org/). We examined the NBK control database (397 healthy individuals) and our in-house whole exome sequencing data (59 subjects), which were described in our previous study^21^.

### Plasmids and cell culture

Human embryonic kidney 293 T cells (HEK293T) and HeLa cells were cultured in Dulbecco’s modified Eagle medium (DMEM)-HG (Invitrogen, Carlsbad, CA, USA) supplemented with 10% (v/v) fetal bovine serum (FBS), 100 U/mL penicillin, and 0.1 mg/mL streptomycin. The mammalian expression plasmids for hBEST1 WT, p.Ala195Val, and p.Trp93Cys were previously described^22^. The hBEST1 p.Ile38Ser mutant plasmids were generated by using PCR-based site-directed mutagenesis. Plasmids were transiently transfected into cells using Lipofectamine Plus (Invitrogen). For electrophysiological experiments, h*BEST1* plasmids without tags were transfected at a 9:1 ratio with a plasmid expressing the green fluorescence protein (pEGFP-N1). An average transfection rate over 90% was confirmed by transfection with a plasmid expressing green fluorescence protein (Supplementary Fig. S3). For surface biotinylation, immunocytochemistry, and immunoblotting, h*BEST1* plasmids containing a Myc-tag were used.

### Electrophysiology in cultured cells

Anion channel activities were measured in HEK293T cells using the whole-cell patch clamp techniques reported previously^22, 35^. Briefly, cells were transferred into a bath mounted on a stage with an inverted microscope (IX-70; Olympus, Tokyo, Japan). Conventional whole-cell clamp was achieved by rupturing the patch membrane after forming a gigaseal. The bath solution was perfused at 5 mL/min. The voltage and current recordings were performed at room temperature (22–25 °C). Patch pipettes with a free-tip resistance of approximately 2–5 MΩ were connected to the head stage of a patch-clamp amplifier (Axopatch-700B; Molecular Devices, Sunnyvale, CA, USA). pCLAMP software v. 10.2 and Digidata-1440A (Molecular Devices) were used to acquire data and apply command pulses. AgCl reference electrodes were connected to the bath via a 1.5% agar bridge containing 3 M KCl solution. Voltage and current traces were stored and analyzed using Clampfit v. 10.2 and Origin v. 8.0 (OriginLab Corp., Northampton, MA, USA). Currents were sampled at 5 kHz. All data were low pass-filtered at 1 kHz.

The bath solution for the whole-cell patch clamp contained 146 mM N-methyl-d-glucamine-Cl (NMDG-Cl), 1 mM CaCl_2_, 1 mM MgCl_2_, 5 mM glucose, and 10 mM HEPES (pH 7.4). The pipette solution contained 148 mM NMDG-Cl, 1 mM MgCl_2_, 3 mM MgATP, 10 mM HEPES, and 5 mM ethylene glycol tetra-acetic acid (EGTA) (pH 7.2).The free Ca^2+^ concentrations of the buffer solutions were fixed to 1 μM by adjusting the Ca^2+^ chelator EGTA (5 mM) and CaCl_2_ concentrations using WEBMAX-C software (http://www.stanford.edu/~cpatton/maxc.html). The osmolarity of the bath solution was set to be 10 mOsm higher than that of the pipette solution by adding sorbitol to suppress the volume-activated anion channels. To determine the current-voltage (I-V) relationship, the clamp mode was shifted to voltage clamp mode, and the I-V curve was obtained by applying step pulses from −100 to 100 mV (voltage interval: 20 mV; duration: 2 s; holding potential: 0 mV).

### Surface biotinylation, immunoblotting, and detergent solubility assay

Surface biotinylation and immunoblotting were performed as described previously^22^. Transfected HEK293T cells were washed three times with ice-cold phosphate-buffered saline (PBS). The cells were then treated with sulfo-NHS-SS-biotin-containing buffer (Pierce, Rockford, IL, USA) for 30 min to biotinylate the plasma membrane proteins. After biotinylation, the cells were washed with quenching buffer to remove the excess biotin and washed twice again with PBS. The cells were harvested and incubated overnight with avidin solution (UltraLink Immobilized NeutrAvidin Beads 10%, Pierce) at 4 °C. Avidin-bound complexes were washed three times and the biotinylated proteins were eluted in a 2X sample buffer. The protein samples were suspended in a sodium dodecyl sulfate (SDS) buffer and separated by SDS-polyacrylamide gel electrophoresis. The separated proteins were transferred to a nitrocellulose membrane and blotted with the appropriate primary and secondary antibodies. The anti-Myc (Santa Cruz Biotechnology, Santa Cruz, CA, USA) antibody was used as the primary antibody, and an anti-mouse IgG (HRP) (Thermo Scientific, Rockford, IL, USA) antibody was used as the secondary antibody. Protein bands were detected by enhanced chemiluminescence (Amersham Biosciences, Buckinghamshire, UK).

Detergent solubility assay was performed as described previously^36^. Transfected HEK293T cells were washed twice with ice-cold PBS and lysed with 0.5% Triton X-100 (Tx) containing lysis buffer for 2 min on ice. After centrifugation, the Tx-soluble fraction was collected and denatured in SDS buffer. The pellet containing Tx-insoluble proteins was sonicated and denatured in SDS buffer containing 9 M urea. The samples were analyzed by western blotting as described previously.

### Immunocytochemistry

Immunocytochemistry was performed as described previously^22^. Cells grown on coverslips and tissue sections were fixed in 10% formalin for 10 min and permeabilized with 0.1% triton X-100 for 10 min at room temperature. Nonspecific binding sites were blocked by incubation for 1 h at room temperature with 0.1 mL of phosphate buffered saline (PBS) containing 5% horse serum, 1% bovine serum albumin, and 0.1% gelatin (blocking medium). After blocking, cells were stained by incubation with appropriate primary antibodies and then treated with fluorophore-tagged secondary antibodies. Fluorescent images were obtained with a Zeiss LSM 780 confocal microscope (Carl Zeiss, Berlin, Germany). Anti-myc (Santa Cruz Biotechnology), and anti-Na^+^-K^+^ ATPase (Abcam, Cambridge, MA, USA) were purchased from commercial sources.

### Structure modeling

Structure modeling was performed as previously described^37, 38^. The template structure that was sequentially most similar to the hBEST1 was selected by using Blast sequence search against PDB. The calcium-activated chloride channel bestrophin-1 from chicken (pdb ID: 4RDQ) was selected. SWISS-MODEL was used to generate tertiary structure of the domain (SWISS-MODEL, http://swissmodel.expasy.org/). Molecular graphics and analyses were performed with the UCSF Chimera package (Chimera, http://www.cgl.ucsf.edu/chimera).

### Statistical analysis

The results are presented as the means ± SEM. Statistical analysis was performed with ANOVA followed by Newman-Keuls multiple comparison test as appropriate. *P* < 0.05 was considered statistically significant.

## Electronic supplementary material

Supplementary information
