# Supplementary material for: Adult-Onset Vitelliform Macular Dystrophy caused by BEST1 p.Ile38Ser Mutation is a Mild Form of Best Vitelliform Macular Dystrophy
Source: Sci Rep. 2017 Aug 22;7:9146. doi: 10.1038/s41598-017-09629-9 (PMC5567297; doi:10.1038/s41598-017-09629-9)
Supplement: Supplementary file 1 — Supplementary information [file 41598_2017_9629_MOESM1_ESM.pdf]

Adult-Onset Vitelliform Macular Dystrophy caused by BEST1 p.Ile38Ser Mutation is a Mild Form of Best Vitelliform Macular Dystrophy

Ikhyun Jun[1,2,3], Joon Suk Lee[3], Ji Hwan Lee[1], Christopher Seungkyu Lee[1], Seung-il Choi[2], Heon Yung Gee[3], Min Goo Lee[3], Eung Kweon Kim[1,2,4,*]

[1]The Institute of Vision Research, Department of Ophthalmology, Yonsei University College of Medicine, Seoul, Korea

[2]Corneal Dystrophy Research Institute, Yonsei University College of Medicine, Seoul, Korea

[3]Department of Pharmacology and Brain Korea 21 PLUS Project for Medical Sciences, Yonsei University College of Medicine, Seoul, Korea

[4]Severance Biomedical Science Institute, Yonsei University College of Medicine, Seoul, Korea

Supplementary Table S1. Detected nonsynonymous variants of *PRPH2*, *IMPG1*, and *IMPG2*. in the subject.

Gene	Nucleotide change[a]	Amino acid change	Frequencies in the dbSNP database[b]	Frequencies in the ExAC database[c]
PRPH2	c.910C>G	p.Gln304Glu	rs390659 (MAF:G=0.2434/1219)	94370/121392 (36804 hom)
	c.929G>A	p.Arg310Lys	rs425876 (MAF:C=0.0587/294)	110687/121410 (50607 hom)
	c.1013A>G	p.Asp338Gly	rs434102 (MAF:T=0.2426/1215)	94027/120706 (36719 hom)
IMPG1	c.1552C>G	p.His518Asp	rs3734311 (MAF:G=0.4651/2329)	56217/120658 (13556 hom)
	c.2377G>A	p.Asp793Asn	rs76604824 (MAF:T=0.0463/232)	1433/38120 (39 hom)
IMPG2	c.2021C>T	p.Thr674Ile	rs571391 (MAF:G=0.3508/1757)	78869/121034 (25908 hom)

[a]cDNA mutations are numbered according to human cDNA reference sequence NM_000322 (*PRPH2*), NM_001563 (*IMPG1*), and NM_016247 (*IMPG2*); +1 corresponds to the A of ATG translation initiation codon. [b]dbSNP database (http://www.ncbi.nlm.nih.gov/SNP). [c]ExAC browser (http://exac.broadinstitute.org/).

Supplementary Table S2. Conductance values of variants, and summary of analysis of variance (ANOVA) followed by Newman-Keuls multiple comparison test of Figure 5E.

**Conductance**	**G_{chord} (nS, mean ± SEM)**	
Mock	0.45 ± 0.10	
WT	25.25 ± 2.08	
p.Ile38Ser	5.20 ± 0.86	
p.Ala195Val	1.78 ± 0.53	
p.Trp93Cys	0.78 ± 0.19	
WT + p.Ile38Ser	10.65 ± 1.80	
WT + p.Ala195Val	19.46 ± 1.99	
WT + p.Trp93Cys	2.97 ± 0.46	
**Newman-Keuls Multiple Comparison Test**	***P* < 0.05?**	**Summary**
Mock vs WT	Yes	***
Mock vs WT + p.Ala195Val	Yes	***
Mock vs WT + p.Ile38Ser	Yes	**
Mock vs p.Ile38Ser	No	ns
Mock vs WT + p.Trp93Cys	No	ns
Mock vs p.Ala195Val	No	ns
Mock vs p.Trp93Cys	No	ns
p.Trp93Cys vs WT	Yes	***
p.Trp93Cys vs WT + p.Ala195Val	Yes	***
p.Trp93Cys vs WT+ p.Ile38Ser	Yes	**
p.Trp93Cys vs p.Ile38Ser	No	ns
p.Trp93Cys vs WT+ p.Trp93Cys	No	ns
p.Trp93Cys vs p.Ala195Val	No	ns
p.Ala195Val vs WT	Yes	***
p.Ala195Val vs WT+ p.Ala195Val	Yes	***
p.Ala195Val vs WT+ p.Ile38Ser	Yes	**
p.Ala195Val vs p.Ile38Ser	No	ns
p.Ala195Val vs WT+ p.Trp93Cys	No	ns
WT+ p.Trp93Cys vs WT	Yes	***
WT+ p.Trp93Cys vs WT+ p.Ala195Val	Yes	***
WT+ p.Trp93Cys vs WT+ p.Ile38Ser	Yes	**
WT+ p.Trp93Cys vs p.Ile38Ser	No	ns
p.Ile38Ser vs WT	Yes	***
p.Ile38Ser vs WT+ p.Ala195Val	Yes	***
p.Ile38Ser vs WT+ p.Ile38Ser	Yes	**
WT+ p.Ile38Ser vs WT	Yes	***
WT+ p.Ile38Ser vs WT+ p.Ala195Val	Yes	***
WT+ p.Ala195Val vs WT	Yes	**

Abbreviations are as follows: SEM, standard error; ns, not significant; WT, wild type; *, $P < 0.05$; **, $P < 0.01$; ***, $P < 0.001$.

Supplementary Table S3. **Primer Designed for Amplification of *PRPH2*, *IMPG1*, and *IMPG2*.**

Gene	Exon no.	Sequence (5’……3’)
PRPH2	Exon 1A F	AGGGCTTCCATCTGGCATACTTG
	Exon 1A R	CTATCCCCTGCTCAAGCTGTGATTC
	Exon 1B F	AAGTTGTGCACCCGATGGAGA
	Exon 1B R	CAGCCTAGGACTGTTCCTGAAGATTG
	Exon 2 F	CCCACAGCTCCACTGAAGGC
	Exon 2 R	CCAAGTGTGCGAGTGAATGACTATTCT
	Exon 3 F	TCAGGGAGAGTCTCTGTAAGATGGT
	Exon 3 R	CAGGTGTGTTGAGCACTGAGGAC
IMPG1	Exon 1 F	TGTTGAATTTCGGTGGATAAATGGA
	Exon 1 R	TCCAGAAACAGACACTGCTACATGTTC
	Exon 2 F	GCTAAAGACAACCTATGGATGAATGA
	Exon 2 R	GCTTGATCGGCTCATTATACTATTCTC
	Exon 3 F	GCCTAATCAGATACCTCCAAGCA
	Exon 3 R	TGTGTCGACATAAGCCATGAAATAA
	Exon 4 F	CACAGCTCTTAGTGGCTGACAT
	Exon 4 R	TGCAAAATAATATGGTACAGTCAGGTTGA
	Exon 5 F	ACATGCTATCATGATGATTTGAATGAAAGTTG
	Exon 5 R	GGTCAATCTAGTTTTAAAGTATGCTTTTCCT
	Exon 6 F	CCCTCAGTACCTAGGAAGAAGTGAGAA
	Exon 6 R	AACACATACAGGGAAGCACCAAA
	Exon 7 F	TGGAACTGTTCGCCGTAAGGG
	Exon 7 R	GCTTCCAAAGGTGGCCCAAA
	Exon 8 F	TCCAGGATTTGGCAGAGCTAAACA
	Exon 8 R	AGGAACAAAGAGTTTGGCCTCCTT
	Exon 9 F	AAAAGTAATGGGCTTATCCAAGACAT
	Exon 9 R	CAGTAAGAGCTGAGAAATCCATTTACA
	Exon 10 F	TTCCTGTCACAGTAATGATCGACTT
	Exon 10 R	TTCTCTCCGAGCCCATCAGGAA
	Exon 11 F	TGGGTTAAGCAAGTGCTGCTTGT
	Exon 11 R	CCAGCCTGCTTGGTCCAAATC
	Exon 12 F	TGGGTTCGGGATGGCTTTGT
	Exon 12 R	GGCAGATTCAGTTATCCGAGAAGCA
	Exon 13A F	GGGCAATGGTCATAGAACTAGTGGTG
	Exon 13A R	TGAAATGATCTACGCAAATGCTATGT
	Exon 13B F	ATGTCTTCAGCAACAGCTGGCTT
	Exon 13B R	CCCCACCAGTGATTATTCTGCAA
	Exon 14 F	AATGATGTGCTCCATAGCTTCCAAA
	Exon 14 R	GGGACTAATCACTGCAAATCAACCA
	Exon 15 F	TGGGTTGAAAGGACCATATGAATTT
	Exon 15 R	TGGAGCTAAACTAATATCACTTGTGTC
	Exon 16 F	ATGTCACCCCCTTAAAACAGTAAATT
	Exon 16 R	TTTCAGCAACCACAACTCAGAACTT
	Exon 17 F	AAATTTTCAGGGAAGGTGGAAGCA
	Exon 17 R	AGAAGATGTCATAAATGGCAAGCA
IMPG2	Exon 1 F	TCCATTTCTTACAGCAATCACATCA
	Exon 1 R	TTCTTAGTGGACTGCTTGTTAAAGG
	Exon 2 F	AGTAGAAAGGTAGTTTTGGCTCAGT
	Exon 2 R	CCTATGATTTGGGCACTGGCTTCT
	Exon 3 F	TTTTGCTTCCTTTAGGCCTTAGC
	Exon 3 R	GAGACACTCCAGCTCATGAAGAGAT
	Exon 4 F	CTTATCCACAGGCCTGGTCATT
	Exon 4 R	TTTGATTCTAAATCCATGGCAGGTT
	Exon 5 F	AAAATGTACTTGTTTGTGAGCCTGTCT
	Exon 5 R	GCAATATTGTGCTAGAGGACTAAGG
	Exon 6 F	TGTAGTCCAGCAATGGGAGATAA
	Exon 6 R	CTCTCTGGTAGAGGAGTTACTTCTTTT

Exon 7 F	TAACCACTCCAGGAACCAAGTAG
Exon 7 R	GCCTGTGTCTTAAAGTCTACCCAAT
Exon 8 F	CCTACGTAACTTGTCAGCAGCAA
Exon 8 R	TTTTCTTTCATTTTCTGACCTGGGTAT
Exon 9 F	GGCAGAATTGAGTAGTTGCAACAGAGA
Exon 9 R	GCCAACAACTGGAGTCCTCTGC
Exon 10 F	TATCAGGTTGGCTCCTGTCTCAT
Exon 10 R	AGTGATGGAATCCATGCTCTTTGAG
Exon 11 F	GTAATGGTCTAGGGATGATGCAAGA
Exon 11 R	CGCTTCATAGGAATCTTGAACAGA
Exon 12 F	TCATAGCCATAGTCTCTTCCTCTCT
Exon 12 R	ATTATTAATACACCGCCTCCTTGTC
Exon 13A F	AAGCCACGGCTTGGACAGTG
Exon 13A R	TGAGTGACTGTCCCATATTGCAAACA
Exon 13B F	AGGGTTAGAGACGCAGATTCAG
Exon 13B R	GTTCATTCACTCAACCTGTGCCAAA
Exon 13C F	TCATCCTCTAGCAGGGTGGAGATT
Exon 13C R	AGCACTCAAAATATGAACATGATGACA
Exon 13D F	TTTCTTACCAACCCTAGGCCATGA
Exon 13D R	GAGAAATTGTCCAGAGACATATTGGCA
Exon 14 F	ATGTTTGGACGATGTTTTGAAAAGG
Exon 14 R	TAGGAAAAGTGAGGCAGGGTCTTAC
Exon 15 F	AAATTATAGGTGCAGGCCCCTTTTC
Exon 15 R	GGTTTTATGATTTGGCTCTGAGTTG
Exon 16 F	GCATCTTATTCTTAGTGCTACTTCTGT
Exon 16 R	AGCTTCACGTGGTCAGCATTTAT
Exon 17 F	ACACTCATACACACCCCCACCC
Exon 17 R	GCCATGGGTGTGAGGAAATCATAA
Exon 18 F	TGTGCTCACTCAGGTGTGACATTA
Exon 18 R	CTCCTAGTCTATGATATGCACAGAGT
Exon 19 F	ATTCCCTTACTAAGCCAGACTTCTCCA
Exon 19 R	GCACAGAACAGAACAACTTTCCTTCA

Supplementary Figure S1.

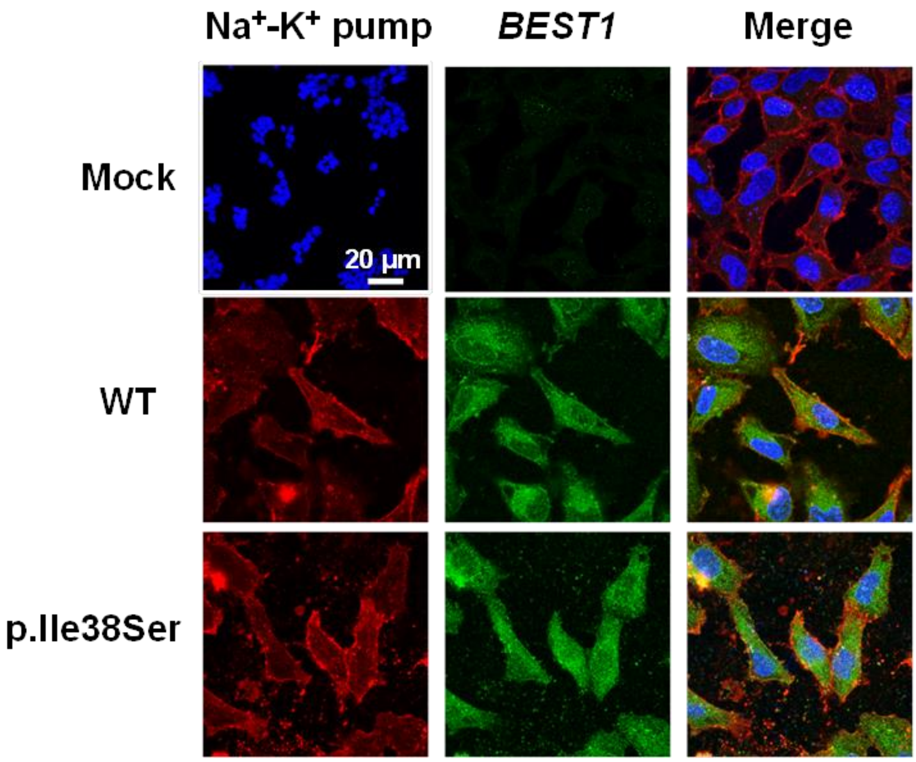

Supplementary Figure S1. Immunocytochemistry of hBEST1 wild type and p.Ile38Ser mutant. Hela cells were transfected with plasmids expressing wild type and p.Ile38Ser hBestrophin-1. hBEST1 (Green) was co-stained with the plasma membrane marker protein Na+-K+ pump (Red) and nucleus marker DAPI (Blue). Surface expression of wild type and mutant hBEST1 was confirmed by immunocytochemistry.

Supplementary Figure S2.

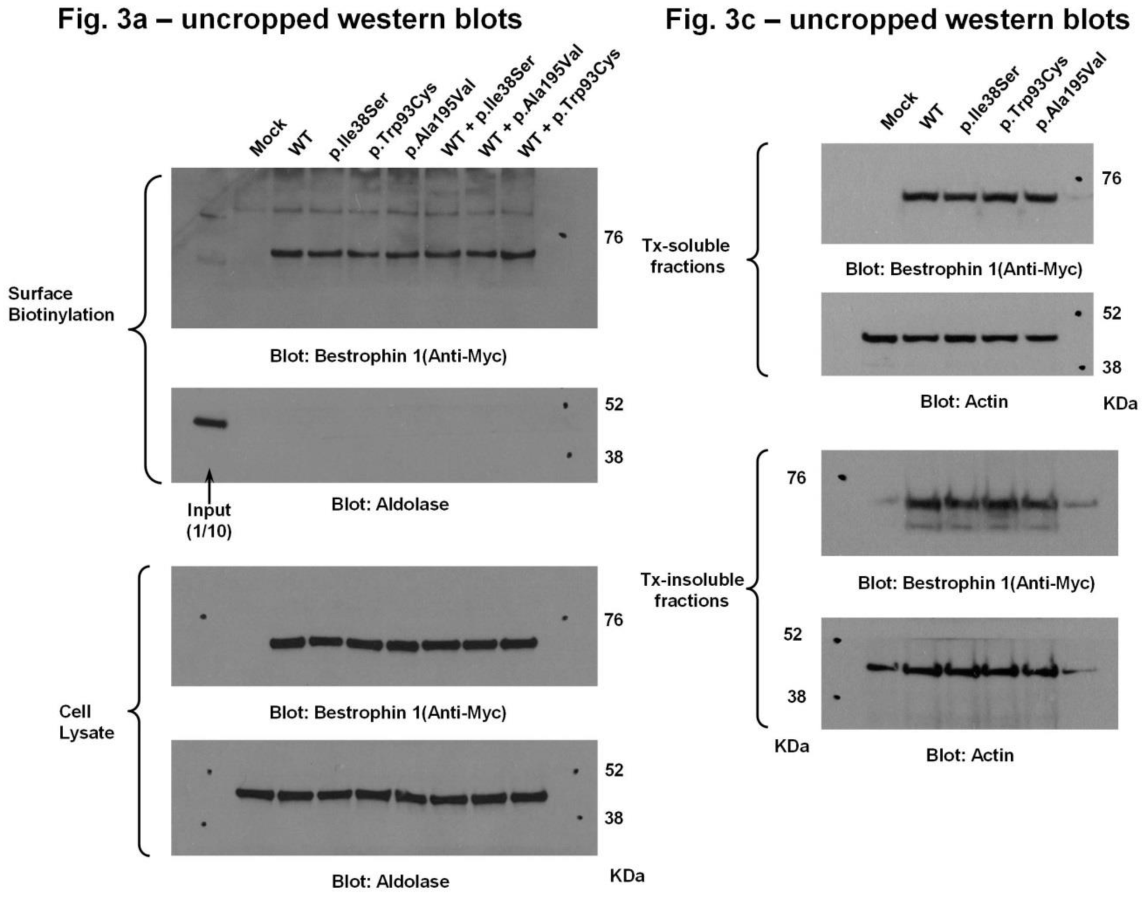

Supplementary Figure S2. Figure showing uncropped western blots from Fig. 3.

Supplementary Figure S3.

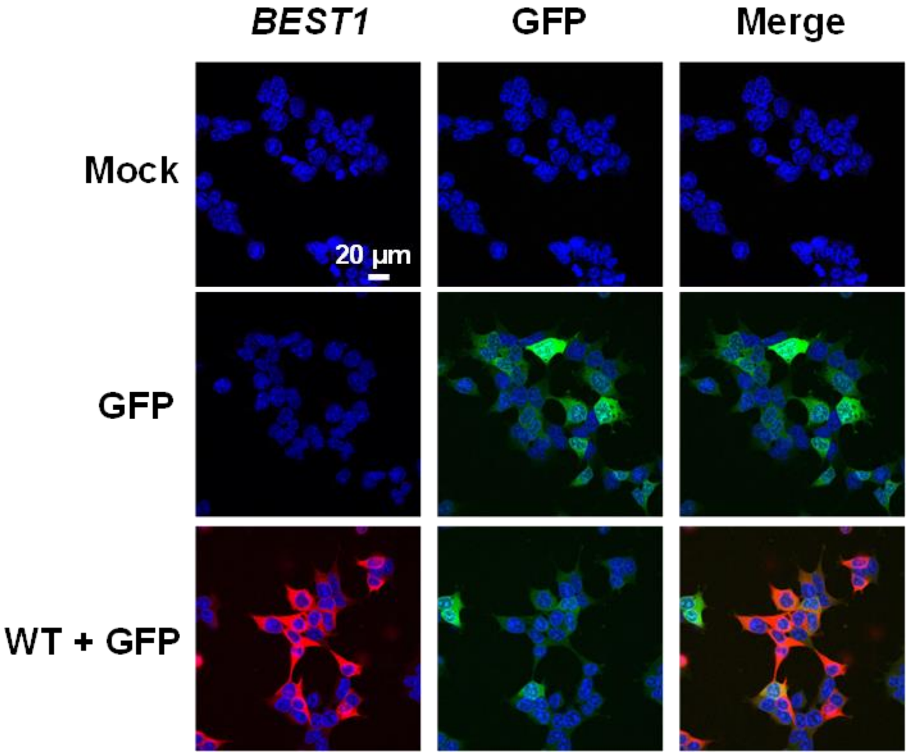

Supplementary Figure S3. Transfection efficiency of HEK293T cells which we used in the electrophysiological experiments. HEK293T cells were transfected with plasmids expressing green fluorescence protein (GFP), and wild type hBestrophin-1. hBEST1 (Red) was stained with nucleus marker DAPI (Blue). Average transfection rate over 90% was confirmed by immunocytochemistry.
